# Characterization of size‐specific effects during dual‐energy CT material decomposition of non‐iodine materials

**DOI:** 10.1002/acm2.13471

**Published:** 2021-11-16

**Authors:** Jessica Miller, Lianna DiMaso, Jessie Huang‐Vredevoogd, Jainil Shah, Michael Lawless

**Affiliations:** ^1^ Department of Human Oncology University of Wisconsin Madison Wisconsin USA; ^2^ Department of Medical Physics University of Wisconsin Madison Wisconsin USA; ^3^ Siemens Medical Solutions USA, Inc. Malvern Pennsylvania USA

**Keywords:** CT simulation, dual‐energy CT

## Abstract

**Purpose:**

The dual‐energy CT (DECT) LiverVNC application class in the Siemens Syngo.via software has been used to perform non‐iodine material decompositions. However, the LiverVNC application is designed with an optional size‐specific calibration based on iodine measurements. This work investigates the effects of this iodine‐based size‐specific calibration on non‐iodine material decomposition and benchmarks alternative methods for size‐specific calibrations.

**Methods:**

Calcium quantification was performed with split‐filter and sequential‐scanning DECT techniques on the Siemens SOMATOM Definition Edge CT scanner. Images were acquired of the Gammex MECT abdomen and head phantom containing calcium inserts with concentrations ranging from 50–300 mgCa/ml. Several workflows were explored investigating the effects of size‐specific dual‐energy ratios (DERs) and the beam hardening correction (BHC) function in the LiverVNC application. Effects of image noise were also investigated by varying CTDI_vol_ and using iterative reconstruction (ADMIRE).

**Results:**

With the default BHC activated, Syngo.via underestimated the calcium concentrations in the abdomen for sequential‐scanning acquisitions, leaving residual calcium in the virtual non‐contrast images and underestimating calcium in the enhancement images for all DERs. Activation of the BHC with split‐filter images resulted in a calcium over‐ or underestimation depending on the DER. With the BHC inactivated, the use of a single DER led to an under‐ or overestimate of calcium concentration depending on phantom size and DECT modality. Optimal results were found with BHC inactivated using size‐specific DERs. CTDI_vol_ levels and ADMIRE had no significant effect on results.

**Conclusion:**

When performing non‐iodine material decomposition in the LiverVNC application class, it is important to understand the implications of the BHC function and to account for patient size appropriately. The BHC in the LiverVNC application is specific to iodine and leads to inaccurate quantification of other materials. The inaccuracies can be overcome by deactivating the BHC function and using size‐specific DERs, which provided the most accurate calcium quantification.

## 1 INTRODUCTION

Application of dual‐energy CT (DECT) material decomposition for radiation therapy has grown over recent years. DECT material decomposition shows promise to inform radiation therapy treatment response,^1^ provide non‐contrast images for dose calculation,^2^ and provide functional imaging to reduce radiation‐induced toxicities to healthy tissue.^3^, ^4^ Iodine quantification has historically been the primary focus of DECT material decomposition. To isolate iodine, many studies use the LiverVNC application within Siemens’ Syngo.via Software which is designed to perform iodine material decomposition in the liver. The accuracy of iodine quantification using the LiverVNC application has been well documented for images acquired on a variety of CT scanner models and DECT techniques.^5^, ^6^, ^7^

As the use of DECT continues to increase, material quantification has expanded to non‐iodine materials such as iron,^8^, ^9^, ^10^, ^11^ calcium,^12^, ^13^, ^14^ and xenon.^15^, ^16^ Non‐iodine material decomposition can be performed in the LiverVNC application by changing the material definitions in the configuration settings. Several studies have investigated non‐iodine material decomposition using the LiverVNC application to identify and quantify these materials. Iron quantification via the LiverVNC application shows potential to quantify hepatic iron accumulation,^8^, ^9^ as well as to quantify liver fat content in the presence of iron and iodinated contrast material.^10^, ^11^ Of particular interest for this work is the removal of calcium for the assessment of active and inactive marrow. The delineation of bone marrow is applicable to radiation therapy, as bone marrow is a highly radiosensitive organ that can be a limiting factor in pelvic radiotherapy plans^17^ or a target for radiotherapy plans that treat cancers in blood‐forming tissues.^12^ The LiverVNC application has been used to create virtual non‐calcium images (VNCa) for bone marrow assessment and identification of traumatic bone lesions in the knee^18^ and ankle joint^13^ as well as radiation‐induced bone marrow edema in the mandible.^14^

While these studies report promise for DECT non‐iodine material decomposition using the LiverVNC application, no work has been published discussing the impact of patient size on the accuracy of these material decompositions. The LiverVNC applications include a default beam hardening correction (BHC) algorithm which is specific to iodine measurements. It is a size‐dependent selection of the parameterization of the material decomposition. In addition, non‐iodine material decomposition requires an input of a dual‐energy ratio (DER) which is also dependent on patient size. The calculation and application of the BHC and DER are interconnected and must be well understood prior to use of the software. Several publications have investigated iodine removal for various phantom sizes using Syngo.via applications with default DERs and BHC activation^5^, ^6^, ^7^; however, the same body of work has not been done for other materials. It is necessary to determine if the iodine‐specific BHC is appropriate for non‐iodine material decomposition, and if not, to determine an accurate method for size correction.

Thus, the goal of this work was to develop a method for non‐iodine material decomposition which accurately accounts for patient size using the LiverVNC application. To achieve this, a series of image processing workflows were investigated in phantom, first using iodine to benchmark the methods of this study against previous work, and then using calcium to determine a methodology for non‐iodine material decomposition.

## 2 METHODS

### 2.1 Phantom

To simulate a range of patient sizes, images were acquired using the Gammex MECT phantom (Gammex Inc, Middleton, Wisconsin, USA). The elliptical, body portion of the phantom was designed to mimic the torso of a larger patient with a diameter of 35 cm and the removable head insert was designed with a diameter of 20 cm.^19^ Gammex MECT inserts with iodine concentrations ranging from 2 to 15 mg/ml and calcium concentrations ranging from 50 to 300 mg/ml were placed in the inner head portion of the phantom for all corresponding image acquisitions.

### 2.2 CT acquisition

Images were acquired with a Siemens SOMATOM Definition Edge CT scanner (Siemens Healthineers, Forchheim, Germany) using two single‐source DECT acquisition techniques, TwinBeam and Dual Spiral. The TwinBeam technique utilizes a split‐filter to create spectral separation from a single 120 kVp beam.^20^ The Dual Spiral technique relies on sequential 140 and 80 kVp acquisitions.^21^ For both techniques, images were acquired with a total CTDIvol of 25 mGy, 3 mm slice thickness, and a 50 cm FOV. The D30 kernel was used for all image reconstructions. The D30 kernel does not include beam hardening corrections for bone.^22^

### 2.3 Image noise investigation

It has been shown that image noise, caused by large body habitus, can lead to inaccuracies in DECT reconstructions.^23^, ^24^ To isolate the effects of noise in size‐induced variations for DECT reconstructions, several additional image acquisitions were performed. To reduce the image noise in the large body scans, the body images were reconstructed with Siemens iterative noise reduction, ADMIRE strength 5. To increase the noise in images of the head phantom, an additional set of images were acquired with reduced mAs. The mAs was reduced so that the image noise in the reduced‐mAs head phantom images matched the image noise measured in the standard body phantom images (acquired with a CTDIvol of 25 mGy). To achieve this image noise match, the mAs was reduced to roughly 10% of the original mAs for both DECT techniques.

### 2.4 Creation of virtual non‐contrast images

All virtual non‐contrast and enhancement images were created with Siemens’ Syngo.via Software, VB30 (Siemens, Germany) using the LiverVNC application. The configuration settings of the LiverVNC application allows for several tunable material definition parameters to perform material decomposition. The system relies on the CT numbers for two basis materials in the high‐ and low‐energy images. The default values correspond to tissue and fat. In addition, two size‐specific parameters are used to quantify a given material, the DER and BHC. The DER, described in Primak et al.^23^ is a density‐independent material‐specific parameter that is highly dependent on the spectral separation between the low‐ and high‐energy beams and the size of the image object. The BHC activates the BHC for iodine contrast enhancement (Siemens’ manual) and can either be activated or deactivated in the configuration settings. To isolate the effects of size‐specific parameters within the software, the DER and BHC were investigated.

To calculate DER values, CT numbers for inserts of various concentrations of iodine and calcium were measured in the low‐ and high‐energy images for each DECT technique in the body and head phantom setups. The DER values were calculated as the slope of the linear fit of the CT numbers from the low‐energy image plotted as a function of the CT numbers from the high‐energy images. DER values for TwinBeam and Dual Spiral are displayed in Table 1. Tissue and fat were assigned as basis materials for iodine material decomposition. Fat and CB2, an epoxy resin,^25^ were used as basis materials for the calcium material decomposition due to the material makeup of the calcium inserts.

**TABLE 1:** Dual‐energy ratio (DER) values calculated for iodine and calcium inserts measured in the head and body phantom for TwinBeam and Dual Spiral

	**DER Iodine**			**DER Calcium**	
	**Head**	**Body**	**Default**	**Head**	**Body**
TwinBeam	1.40	1.30	1.46	1.19	1.14
Dual Spiral	1.97	2.18	2.00	1.52	1.57

For each DECT technique, eight workflows were investigated to create virtual non‐contrast images for iodine (VNC) and calcium (VNCa). To isolate the effects of DER and BHC on size‐specific variations during material decomposition, images were created with all possible combinations of activating or deactivating the BHC, applying the head or body DER values, and acquiring DECT images of the head or body phantom setups. Table 2 provides a summary of each workflow.

**TABLE 2:** Workflows used to create virtual non‐contrast (VNC and VNCa) images. The table indicates if the iodine beam hardening correction (BHC) was activated, states the phantom setup for calculation of the dual‐energy ratio (DER) values, and the phantom setup imaged for creation of non‐contrast images

**Workflow**	**Iodine BHC**	**DER value**	**Phantom size**
1	Active	Head	Head
2	Active	Head	Body
3	Active	Body	Head
4	Active	Body	Body
5	Inactive	Head	Head
6	Inactive	Head	Body
7	Inactive	Body	Head
8	Inactive	Body	Body

### 2.5 Virtual enhancement images and concentration calculations

Although the focus of this work was accurate virtual non‐contrast imaging, the impact of errors in VNC and VNCa images naturally impact the CT numbers present in the enhancement images and thus the calculated concentrations. With both iodine and calcium enhancement images, a size‐specific calibration curve is required to calculate concentration (mg/ml) from CT number (HU). For the purpose of this discussion, calibration curves calculated using the Dual Spiral workflows 5 and 8 were applied to all eight workflows. The workflow 5 calibration curve was applied to all head phantom images (workflows 1, 3, 5, and 8) and the workflow 8 calibration curve was applied to all body phantom images (workflows 2, 4, 6, and 8). This method will henceforth be referred to as the manual method.

To benchmark this methodology, iodine concentrations, in mg/ml, were also calculated within the LiverVNC application using the default calibration applied by the software. This method will be referred to as the Syngo.via method. The LiverVNC application does not provide concentration values for non‐iodine materials. As such, the iodine results for the Syngo.via method were compared to those of the manual method to validate a methodology, which could be applied to non‐iodine materials. Concentration values were calculated in the LiverVNC application using a manually placed ROI on the central slice within the phantom. The Syngo.via calculated iodine values were compared to the manual iodine concentrations.

### 2.6 Image analysis

For all virtual non‐contrast and enhancement images, the mean CT number and standard deviation in each insert were calculated by placing a circular ROI in the center of the insert. The ROI was placed in the central slice within the phantom with a diameter of roughly 60% of the cross‐sectional diameter of the insert. The ROI was copied over several slices.

The data that support the findings of this study are available from the corresponding author upon reasonable request.

## 3 RESULTS

### 3.1 Iodine virtual non‐contrast images

Based on the material composition of the iodine inserts, accurate removal of the iodine within the inserts would leave behind water‐equivalent material with a CT number of 0 HU. Thus, a value of 0 HU within the inserts would signify that the correct amount of iodine was identified and removed in the creation of the VNC image. The mean CT numbers (HU) within each iodine insert calculated for each VNC images workflow are shown in Table 3.

**TABLE 3:** Mean CT numbers (HU) calculated within iodine inserts in non‐contrast images. Iodine concentrations ranging from 2 to 15 mg/ml were placed within the head and body phantom as indicated in the table. VNC images were reconstructed for each variation of the beam hardening correction (BHC) active and inactive and dual‐energy ratios (DER) measured with the head and body phantom. Data calculated from Dual Spiral and TwinBeam data is displayed in the table

				**CT number (HU) in the VNC image**				
**Iodine BHC**	**DER**	**Phantom size**	**Workflow number**	**2 mg/ml**	**5 mg/ml**	**10 mg/ml**	**15 mg/ml**	**Largest Error (HU)**
Dual Spiral								
Active	Head	Head	1	−1.9	4.6	0.3	2.1	4.6
		Body	2	0.8	3.9	4.7	3.6	4.7
	Body	Head	3	6.4	25.0	39.8	60.6	60.6
		Body	4	6.2	17.6	31.4	42.9	42.9
Inactive	Head	Head	5	−3.2	1.8	−4.9	−5.8	−5.8
		Body	6	−7.3	−16.7	−35.4	−55.1	−55.1
	Body	Head	7	5.1	23.0	36.4	55.2	55.2
		Body	8	−0.7	2.3	3.4	2.4	3.4
TwinBeam								
Active	Head	Head	1	−12.6	−12.8	−25.4	−26.5	−26.5
		Body	2	−1.6	−8.1	−55.2	−38.8	−55.2
	Body	Head	3	−30.9	−54.6	−105.4	−141.6	−141.6
		Body	4	−35.8	−100.4	−272.1	−314.6	−314.6
Inactive	Head	Head	5	−11.0	−9.0	−18.2	−16.5	−18.2
		Body	6	12.3	29.6	32.4	72.2	72.2
	Body	Head	7	−27.6	−47.0	−90.7	−121.1	−121.1
		Body	8	4.5	8.8	−16.5	9.9	−16.5

Within each DECT modality, four of the eight workflows produced VNC images with CT numbers closest to 0 HU in the iodine inserts: workflows 1, 2, 5 and 8. Workflows 1 and 2 applied the head DER for both phantom sizes with the BHC activated. Workflows 5 and 8 deactivated BHC and used size‐specific DER values, meaning the head DER was used for the head phantom images and the body DER was used for the body phantom images.

The remaining four workflows resulted in significantly larger errors. Workflows 3 and 4 applied the body DER to both phantom sizes with the BHC activated, and workflows 6 and 8, which in the absence of the BHC, applied the head DER to the body phantom and the body DER to the head phantom. For each of these workflows, the errors increased with increasing iodine concentration.

### 3.2 Calcium virtual non‐contrast images

The expected CT number within the calcium inserts, in the absence of calcium, was 55 HU. This value was obtained from a measurement of a CB2 insert without calcium. A value of 55 HU in the VNCa image, would signify that all of the calcium was removed while accurately preserving the CT numbers of the remaining CB2 material. The mean CT number (HU) calculated in the VNCa images are shown in Table 4.

**TABLE 4:** Mean CT numbers (HU) calculated within calcium inserts in non‐contrast images. Calcium concentrations ranging from 50 to 300 mg/ml were placed within the head and body phantom as indicated in the table. VNCa images were calculated for each variation of the beam hardening correction (BHC) active and inactive and dual‐energy ratios (DER) measured with the head and body phantom. Data calculated from Dual Spiral and TwinBeam data is displayed in the table

				**CT number (HU) in the VNCa image**			
**Iodine BHC**	**DER**	**Phantom size**	**Workflow number**	**50 mg/ml**	**100 mg/ml**	**300 mg/ml**	**Largest Error (HU)**
Dual Spiral							
Active	Head	Head	1	54.2	54.1	62.1	7.1
		Body	2	72.3	99.4	181.5	126.5
	Body	Head	3	63.8	72.8	116.8	61.8
		Body	4	77.7	109.6	211.2	156.2
Inactive	Head	Head	5	54.6	55.7	55.0	0.7
		Body	6	38.7	36.0	−3.2	−58.2
	Body	Head	7	64.5	75.0	112.2	57.2
		Body	8	48.6	54.8	51.5	−6.4
TwinBeam							
Active	Head	Head	1	29.6	20.9	−3.9	−58.9
		Body	2	−165.4	−423.5	−1024.0	−1079
	Body	Head	3	−22.5	−73.7	−267.8	−322.8
		Body	4	3071	3071	3071	3016
Inactive	Head	Head	5	39.7	39.3	44.8	−15.7
		Body	6	84.6	95.6	206.9	151.9
	Body	Head	7	−3.9	−39.9	−173.5	−228.5
		Body	8	57.1	38.5	51.5	−16.5

Accurate CT numbers were detected in the VNCa images with only three of the workflows, workflows 1, 5 and 8. Just as with the iodine results, workflows 5 and 8 deactivated the BHC and manually accounted for patient size through the use of size‐specific DER values. Workflow 1 activated the BHC and applied the head DER to the head phantom. Since the BHC corrects for changes in size beyond a head‐sized phantom, the workflow required no size correction and therefore the BHC did not impact the results.

Other than workflow 1, which required no size correction, there were no workflows with the BHC activated that produce accurate VNCa results. Errors tended to increase with increasing calcium concentration. With the BHC deactivated, the use of non‐size‐specific DERs, workflows 6 and 7, also lead to large errors in VNCa images.

### 3.3 Effects of image noise on size‐specific variations

Measurements performed to isolate noise demonstrated only small changes in calcium quantification as a function of noise which do not account for the large variations in calcium quantification with phantom size. The abdomen scans reconstructed using ADMIRE 5 resulted in a noise reduction of 40%–50% for the non‐contrast images. However, the difference between the measured mean values in the standard reconstruction and ADMIRE 5 was less than 3 HU for both DECT techniques. Similarly, reducing the mAs for the head phantom acquisitions demonstrated noise values on par with the abdomen scans, but the mean CT numbers did not vary significantly, with changes in the average HU value of less than 6 HU.

### 3.4 Iodine virtual enhancement images and concentration calculations

Iodine concentration values were calculated using the manual method and the default Syngo.via calibration for the Dual Spiral enhancement images. The concentration values calculated with both methods were in good agreement, with an average difference of 0.1 mg/ml and a maximum difference of 0.9 mg/ml for all concentrations and across all workflows.

**FIGURE 1:**
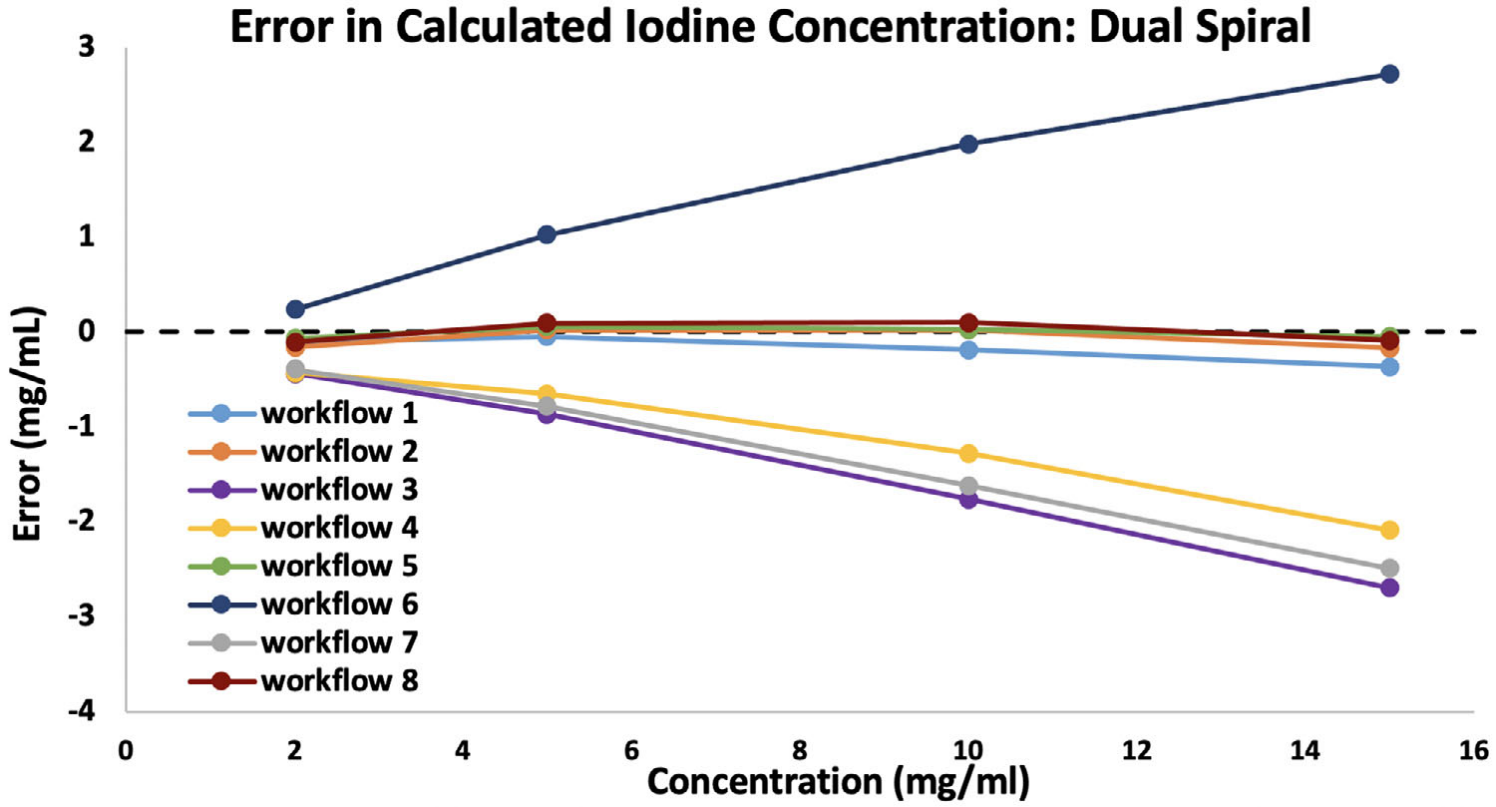
Errors in iodine concentration (mg/ml) for four concentrations of iodine; 2 , 5 , 10, and 15 mg/ml. Errors were calculated from Dual Spiral contrast images calculated with the Syngo software LiverVNC application using eight workflows applying different variations of beam hardening correction (BHC) activation and dual‐energy ratio (DER) values. Concentrations were determined using the manual method. Details for each workflow can be found in Table 2

The errors in iodine concentration calculated with the manual method are shown in Figure 1. The errors in the VNC images correlated well with the errors in calculated concentration for each workflow. Workflows 1, 2, 5, and 8 which produced accurate non‐contrast values, also produced a minimal error in iodine concentration, with a maximum error of −0.5 mg/ml. Workflows 3, 4, and 7, which produced non‐contrast images with high HU values, underestimated iodine in the enhancement images by 17%, on average, across all concentrations. Workflow 6 resulted in low HU values in the non‐contrast image and an overestimation of iodine in the enhancement images.

### 3.5 Calcium virtual enhancement images and concentration calculations

**FIGURE 2:**
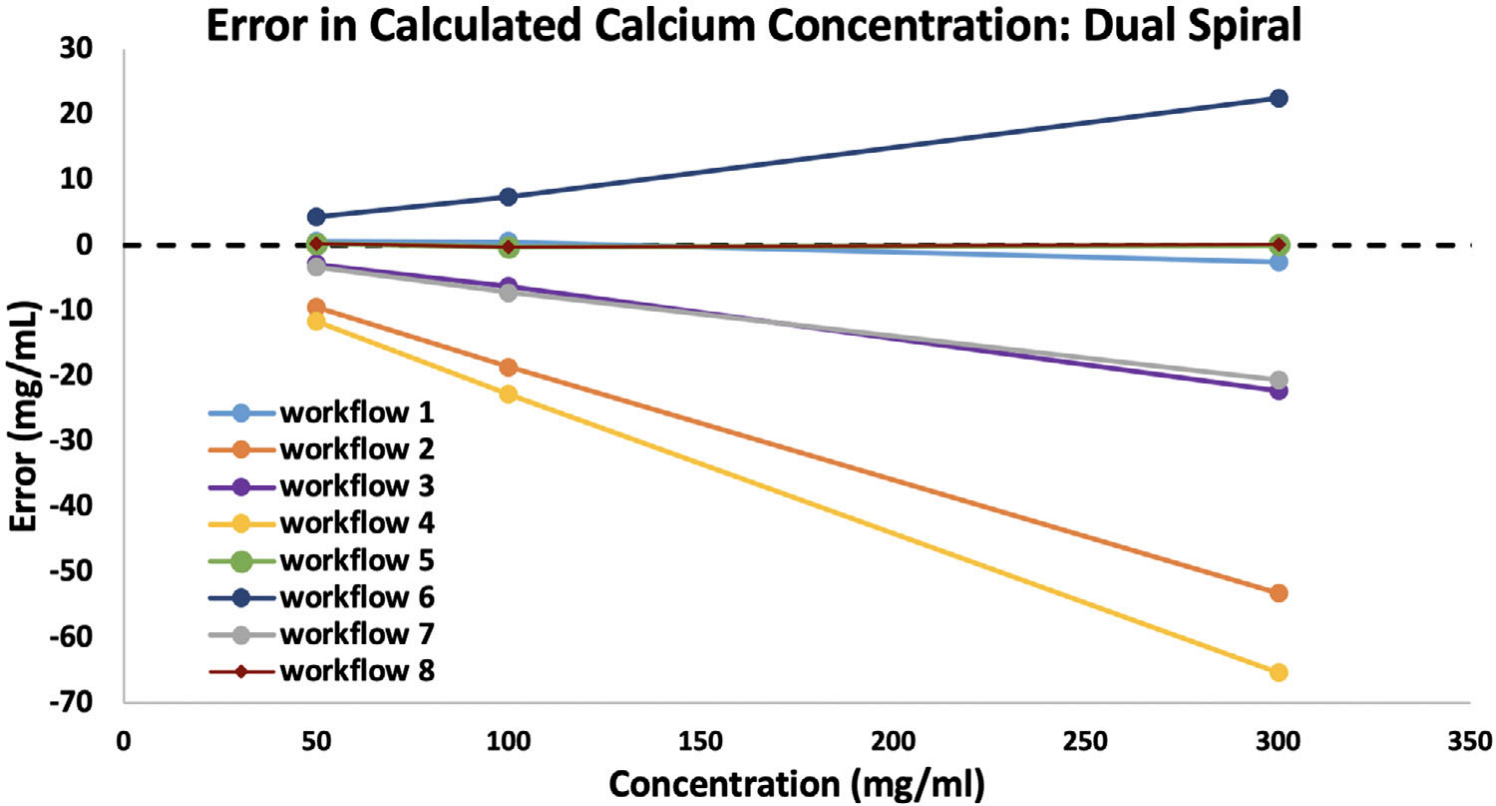
Errors in calcium concentration (mg/ml) for three concentrations of calcium; 50, l00, and 300 mg/ml. Errors were calculated from Dual Spiral contrast images calculated with the Syngo software LiverVNC application using eight workflows applying different variations of beam hardening correction (BHC) activation and dual‐energy ratio (DER) values. Concentrations were determined using the manual method. Details for each workflow can be found in Table 2

A similar trend was demonstrated with the calcium inserts as demonstrated in Figure 2. All workflows which produced errors in the VNCa images, demonstrated similarly scaled errors in the enhancement images, and therefore calculated concentration values. Only workflows 1, 5, and 8 produced accurate concentration values to within 2.6 mg/ml for all three concentrations. As with the iodine results, workflows 3, 4, and 7 underestimated calcium concentrations and workflow 6 overestimated calcium concentrations. Unlike with the iodine results, workflow 2 underestimated calcium concentrations. This is mimicked in the VNCa images with demonstrated high HU values for these workflows.

## 4 DISCUSSION

Use of the BHC and use of size‐specific DERs are both ways that can be used to account for size‐specific influences when performing material decomposition in the Siemens Syngo.via software. However, using an incorrect combination of these two parameters can lead to over or undercorrection of patient size effects and ultimately errors in the resulting material and non‐contrast images. For both iodine and calcium measurements, errors in the non‐contrast images resulted in corresponding errors in the enhancement images, as expected as these two image sets are complementary to one another. For example, when the amount of contrast material is overestimated, the resulting virtual non‐contrast images display low HU values and the corresponding enhancement images display high HU values as is seen for both iodine and calcium using workflow 6 for the Dual Spiral acquisition.

Two of the workflows that produced the most accurate iodine concentration and VNC results, workflows 1 and 2, used the default configurations settings in the LiverVNC application and therefore represent the workflows used in most iodine‐related literature studies (BHC activated, head DER value applied to both head and body size phantoms). These results indicate that for iodine, the BHC accurately accounts for patient size effects and therefore the use of a size‐specific DER is not needed, that is, the use of a single value for the DER, as determined in a head phantom, is sufficient for accurate material decomposition. It is important to note that the BHC factor is designed to correct for a DER calculated from a head‐sized phantom and was also designed specifically to be used for iodine quantification. The iodine DER values measured for this work in the head phantom, 1.40 and 1.97 for TwinBeam and Dual Spiral, respectively, agreed well with the default Syngo.via values at 1.46 and 2.0. Consequently, using the Body DER with the BHC activated led to errors in the VNC and enhancement images for both the head and body phantoms.

The iodine concentrations calculated in this work agreed well with a DECT inter‐manufacturer comparison study performed by Jacobsen et al.^26^ calculated the iodine bias (IB) and total iodine error for iodine inserts (concentrations of 2, 5, and 15 mg/ml) in the MECT body phantom for a split‐filter and sequential scan system. For the sake of comparison, the IB and total iodine error were similarly calculated for this work. The Dual Spiral system produced an IB and total iodine error of −0.1 and 0.17 mg/ml ± 0.11, respectively, compared to −0.78 and 0.75 mg/ml ± 0.13, respectively, for the sequential scan system in Jacobsen et al. The TwinBeam data also showed good agreement, with IB and total iodine errors of −1.2 and 0.87 mg/ml ± 0.39, respectively, for this work, and −2.60 and 1.70 mg/ml ± 0.22, respectively for Jacobsen et al. This agreement for the iodine calculations provides confidence in the methodology presented in this work and consequently applied to calcium data.

**FIGURE 3:**
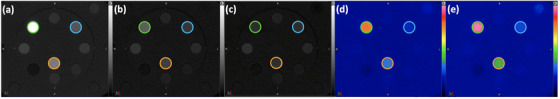
A (a) mixed 120 kVp‐equivalent image, (b) VNCa image (beam hardening corrrection (BHC) active), (c) VNCa image (BHC inactive), (d) calcium image (BHC active) (e) calcium image (BHC inactive). All images were acquired via Dual Spiral CT with the body MECT phantom containing three calcium inserts with calcium concentrations of 300 mg/ml (green circle), 100 mg/ml (orange circle), and 50 mg/ml (blue circle). All material decompositions were performed with a dual‐energy ratio (DER) measured in the body phantom.The window and level is set to 850 and 325 HU, respectively, for all images

When the DER in the LiverVNC application was altered to perform calcium removal, the default setting of having the BHC activated did not produce accurate results for the body phantom (workflow 2). The BHC, which is designed for iodine measurements, underestimated the amount of calcium in each insert, leaving significant quantities of calcium in the VNCa images and underestimating calcium concentrations in the enhancement images by as much as 18.9% for the Dual Spiral system. This concept can be visualized in Figure 3 which shows VNCa and calcium images calculated for the body phantom containing calcium inserts. With the BHC activated the system underestimates the calcium concentration in the calcium images (Figure 3d), producing VNCa images with inaccurately high CT numbers (Figure 3b). This is best visualized in the 300 mg/ml insert (green circle), which is brighter in the VNCa with the BHC activate (Figure 3b) versus inactivated (Figure 3c). The corresponding degradation in CT numbers can be seen in the 300 mg/ml insert in the calcium image with the BHC activated (Figure 3d) versus inactivated (Figure 3e). Therefore, in order to accurately account for patient size when performing non‐iodine material decomposition, the BHC should be deactivated as it is not accurate for materials other than iodine and instead size‐specific DERs should be applied to account for patient size effects.

Many of the published studies using the LiverVNC application for non‐iodine material decomposition do not discuss the size of the phantom used to measure DER values or the use of the BHC. Therefore, it is possible that the results of these studies could be improved further using size‐specific DERs with the BHC deactivated; however, we cannot make definitive statements regarding size‐specific errors without understanding the methods used in each study. It is worth noting that several of these studies investigate non‐iodine material decomposition in the head^14^ or extremities^13^, ^18^ where the object size is relatively small. The effects of the BHC are less significant with small phantom/patient sizes, since the algorithm corrects for size changes with a head‐sized object as reference. The calcium bias, CB, and total calcium error, respectively, for a head phantom using the default settings are −1.5 and 2.7 mg/ml ± 1.8 for the Dual Spiral system and 38.6 and 26.4 mg/ml ± 10.0 for the TwinBeam systems as compared to much larger values of −81.3 and 57.2 mg/ml ± 23.1 for the Dual Spiral system and 874.9 and 611.7 mg/ml ± 244.0 for the TwinBeam systems in the body phantom. In other words, the impact of using this system incorrectly may be minimal for smaller objects and this should be considered when evaluating literature which does not adequately state the Syngo.via settings used during material decomposition.

This work made use of the TwinBeam and Dual Spiral modalities; however, the Syngo.via software is used for all Siemens dual‐energy processing, regardless of the acquisition technique, and thus the findings are of consequence for multiple hardware systems. Additional work should be done with other DECT modalities including high‐energy beams with tin filtration. While a similar trend is expected with other DECT systems, the magnitude of the effect of size‐specific configurations will vary with beam energies and spectral separation. Additionally, two phantom sizes were investigated in this work, which spanned the range of a head and abdomen phantom. More work is needed to understand the impact of size‐specific DER values and patient size for different DECT technologies. While this work provides a methodology for size‐specific, calcium material decomposition using the LiverVNC application, this methodology is applicable to other non‐iodine materials. The magnitude of the errors and uncertainty in measurements from material decomposition images for other materials using the default workflow in the Syngo.via software should be assessed to understand the results of previous work. It is also important to note, that different applications within Syngo.via account for size differences, and these configurations should be well understood prior to use.

Though this study is specific to the material decomposition parameters within the Siemens Syngo.via software, the influence of patient size is nonetheless a factor that can impact dual‐energy images that are generated with other software platforms. This study highlights the importance of understanding the underlying assumptions and size‐specific parameters that go into the generation of material enhancement images and virtual non‐contrast images, as these parameters can have a significant impact on the accuracy of the resulting images. We also present a possible methodology to identify the most accurate configuration for non‐iodine material decomposition that can be applied to other software platforms using a commercially available multi‐energy phantom.

## 5 CONCLUSIONS

When performing non‐iodine material decomposition in the LiverVNC application class in Syngo.via, it is important to understand the implications of the BHC function and to account for patient size appropriately. In addition, it is important that the settings used during material decomposition are clearly stated in the literature so that readers can assess the associated uncertainties of the results. The BHC in the LiverVNC application is specific to iodine and leads to inaccurate quantification of other materials. The inaccuracies can be overcome by deactivating the BHC function and using size‐specific DERs, which provided the most accurate calcium quantification. This workflow can be applied to other non‐iodine materials.

## AUTHOR CONTRIBUTIONS

Dr. Jessica Miller contributed substantially to the conception and design, acquisition of the data, analysis, interpretation of data, was involved in the drafting and revising of the manuscript and has given final approval of the version submitted. Dr. Lianna DiMaso contributed substantially to the conception and design, acquisition of the data, analysis, interpretation of data, was involved in the drafting and revising of the manuscript and has given final approval of the version submitted. Dr. Jessie Huang‐Vredevoogd contributed substantially to the conception and design, was involved in the drafting and revising of the manuscript and has given final approval of the version submitted. Dr. Jainil Shah contributed substantially to the conception and design, was involved in the drafting and revising of the manuscript and has given final approval of the version submitted. Dr. Michael Lawless contributed substantially to the conception and design, acquisition of the data, analysis, interpretation of data, was involved in the drafting and revising of the manuscript and has given final approval of the version submitted.

## Supporting information

Supporting Information
