# Supplementary material for: Characterization of size‐specific effects during dual‐energy CT material decomposition of non‐iodine materials
Source: J Appl Clin Med Phys. 2021 Nov 16;22(12):168–76. doi: 10.1002/acm2.13471 (PMC8664138; doi:10.1002/acm2.13471)
Supplement: Supplementary file 1 — Supporting Information [file ACM2-22-168-s001.docx]

**Characterization of size-specific effects during dual-energy CT material decomposition of non-iodine materials**

Jessica Miller1,2, Lianna DiMaso1, Jessie Huang-Vredevoogd1,2, Jainil Shah^3^, Michael Lawless1

1Department of Human Oncology, University of Wisconsin, Madison, Wisconsin, USA

2Department of Medical Physics, University of Wisconsin, Madison, Wisconsin, USA

3Siemens Medical Solutions USA, Inc., Malvern, PA, USA

**Corresponding Author:**

Jessica Miller, Ph.D., DABR

Associate Professor

University of Wisconsin-Madison

Department of Human Oncology

School of Medicine & Public Health

600 Highland Ave, K4/B86

Madison, Wisconsin 53792

**Running Title:** Size-effects during non-iodine DECT material decomposition

**Key Word:** Dual-Energy CT, CT Simulation

**Acknowledgment:**

Dr. Jessica Miller contributed substantially to the conception and design, acquisition of the data, analysis, interpretation of data, was involved in the drafting and revising of the manuscript and has given final approval of the version submitted. Dr. Lianna DiMaso contributed substantially to the conception and design, acquisition of the data, analysis, interpretation of data, was involved in the drafting and revising of the manuscript and has given final approval of the version submitted. Dr. Jessie Huang-Vredevoogd contributed substantially to the conception and design, was involved in the drafting and revising of the manuscript and has given final approval of the version submitted. Dr. Jainil Shah contributed substantially to the conception and design, was involved in the drafting and revising of the manuscript and has given final approval of the version submitted. Dr. Michael Lawless contributed substantially to the conception and design, acquisition of the data, analysis, interpretation of data, was involved in the drafting and revising of the manuscript and has given final approval of the version submitted.
